# Angiotensin-(1–7) Receptor Mas Deficiency Does Not Exacerbate Cardiac Atrophy Following High-Level Spinal Cord Injury in Mice

**DOI:** 10.3389/fphys.2020.00203

**Published:** 2020-03-12

**Authors:** Anne Järve, Fatimunnisa Qadri, Mihail Todiras, Shirley Schmolke, Natalia Alenina, Michael Bader

**Affiliations:** ^1^Max Delbrück Center for Molecular Medicine, Helmholtz Association of German Research Centers, Berlin, Germany; ^2^Partner Site Berlin, German Center for Cardiovascular Research, Berlin, Germany; ^3^Nicolae Testemiţanu State University of Medicine and Pharmacy, Chişinãu, Moldova; ^4^Charité Universitätsmedizin Berlin, Berlin, Germany; ^5^Institute for Biology, University of Lübeck, Lübeck, Germany

**Keywords:** renin–angiotensin system, cardiac atrophy, atrogenes, sympathetic nervous system, fibrosis

## Abstract

Experimental spinal cord injury (SCI) causes a morphological and functional deterioration of the heart, in which the renin–angiotensin system (RAS) might play a role. The recently discovered non-canonical axis of RAS with angiotensin-(1–7) and its receptor Mas, which is associated with cardioprotection could be essential to prevent damage to the heart following SCI. We investigated the cardiac consequences of SCI and the role of Mas in female wild-type (WT, *n* = 22) and mice deficient of Mas (*Mas^–/–^*, *n* = 25) which underwent spinal cord transection at thoracic level T4 (T4-Tx) or sham-operation by echocardiography (0, 7, 21, and 28 days post-SCI), histology and gene expression analysis at 1 or 2 months post-SCI. We found left ventricular mass reduction with preserved ejection fraction (EF) and fractional shortening in WT as well as *Mas^–/–^* mice. Cardiac output was reduced in *Mas^–/–^* mice, whereas stroke volume (SV) was reduced in WT T4-Tx mice. Echocardiographic indices did not differ between the genotypes. Smaller heart weight (HW) and smaller cardiomyocyte diameter at 1 month post-SCI compared to sham mice was independent of genotype. The muscle-specific E3 ubiquitin ligases Atrogin-1/MAFbx and MuRF1 were upregulated or showed a trend for upregulation in WT mice at 2 months post-SCI, respectively. Angiotensinogen gene expression was upregulated at 1 month post-SCI and angiotensin II receptor type 2 downregulated at 2 month post-SCI in *Mas^–/–^* mice. *Mas* was downregulated post-SCI. Cardiac atrophy following SCI, not exacerbated by lack of *Mas*, is a physiological reaction as there were no signs of cardiac pathology and dysfunction.

## Introduction

Cardiovascular dysfunction is one of the leading causes of morbidity and mortality in both acute and chronic stages of spinal cord injury (SCI) (Weaver et al., 2012). The threefold higher risk of heart disease in people with SCI (Cragg et al., 2013) is caused by the development of cardiovascular risk factors, but might also be caused by direct changes in the heart. Acute heart failure is evident immediately after SCI in autopsy as well as in experimental animals as electron micrographs show signs of calcium excitotoxicity following massive norepinephrine release (Sharov and Galakhin, 1984). Recent studies in a chronic thoracic-level 3 (T3) rat model of SCI showed sustained cardiac atrophy, systolic dysfunction, reduced contractility, as well as a trend for increased myocardial collagen deposition (West et al., 2014; Poormasjedi-Meibod et al., 2019). A meta-analysis of 22 studies with 474 participants found reductions to left ventricle (LV) stroke volume (SV), end-diastolic volume, and LV mass in high-thoracic SCI patients vs. able-bodied. However, in contrast to the experimental data, hearts of SCI patients seem to adapt relatively well to the situation post-SCI as ejection fraction (EF) and diastolic function (Williams et al., 2019) as well as contractility (Driussi et al., 2014) remain preserved. Further research on experimental SCI models could shed light on the discrepancies between human situation and pre-clinical models.

Reduced cardiac weight (atrophy) following cervical or high-thoracic SCI, a consistent finding between studies, is caused by loss of sympathetic innervation to the heart and/or unloading following SCI. In thoracic injuries above T6, control of brainstem over vasculature of the lower body is lost. However, the proportion of loss of the sympathetic input to the heart and the vasculature of the upper thoracic segments depends from the exact level of the SCI. For example, after transection of the spinal cord at T4 (T4-Tx) the heart would lose approx. three-fourth of its sympathetic input, majority of which comes from stellate ganglia (Strack et al., 1988; Lujan and Dicarlo, 2014). Cardiac atrophy is mediated by activation of the ubiquitin–proteasome system and the autophagy–lysosomal machinery (Zaglia et al., 2013; Poormasjedi-Meibod et al., 2019). Its pathological or physiological nature remains to be determined. The renin–angiotensin system (RAS) which usually regulates blood pressure and fluid balance was proposed to be involved in pathological atrophy of the heart following SCI (Poormasjedi-Meibod et al., 2019). This is an interesting possibility, as RAS affects hemodynamics after SCI as we have recently shown (Jarve et al., 2018, 2019), and generates peptide hormones with great impact on the heart (Bader, 2013). The recently discovered non-canonical axis of the RAS including angiotensin-(1–7) which acts via the G protein-coupled receptor (GPCR) Mas exert mostly opposite functions to the classical RAS (Bader et al., 2018) and the Ang-(1–7)-Mas axis has been shown to play a key role in the maintenance of the structure and function of the heart (Santos et al., 2006). For example, an acute treatment with Ang-(1–7) before ischemia/reperfusion injury led to significant recovery of heart function following ischemia in hearts isolated from spinal cord injured rats (Benter et al., 2011). The main actions of Ang-(1–7) in the heart are regulation of genes involved in fibrosis in cardiac fibroblasts via Mas, NO-releasing, antioxidative, NO-increasing, direct anti-hypertrophic effects on cardiomyocytes and regulation of endothelial function (Santos et al., 2006; Rabelo et al., 2008; Bader, 2013). Ang-(1–7)-Mas receptor axis attenuates cardiac remodeling and fibrosis in post-myocardial infarction (Wang et al., 2017). Based on beneficial effects of Mas, we hypothesized that mice lacking Mas have much poorer outcome in the heart when investigated by echocardiography, immunohistochemistry, and molecular biology methods.

## Materials and Methods

### Mice and SCI

Experiments were conducted according to the National Institutes of Health Guide for the Care and Use of Laboratory Animals, and the protocols were approved by the local Animal Care and Use Committee from Berlin LAGeSo (G0132/14). All mice were housed in groups of four to six animals in cages with nesting material, mouse lodges and open access to water and food, at 23°C with a 12 h/12 h circadian cycle. Experiments were performed using wild type (WT, *Mas*^+/+^) vs. *Mas^–/–^* female mice both on C57Bl/6 N background (Walther et al., 1998), weighing 20–25 g, and at about 3 months of age. The experimental plan is depicted under Supplementary Figure 1.

Spinal cord injury was done under intraperitoneal Ketamine (100 mg/kg) – Xylazine (10 mg/kg) anesthesia in combination with isoflurane (1.5–1.8%) inhalation as described previously (Järve et al., 2019). Briefly, a dorsal midline incision was made in the superficial muscle overlying the C7–T3 vertebrae. The dura was opened at the T2–T3 intervertebral gap and the spinal cord was completely transected at thoracic spinal cord segment 4 using microscissors. Complete transection was confirmed by pulling a needle twice between the rostral and caudal spinal cord stumps. Gelfoam was placed above spinal cord to achieve hemostasis. The muscle and skin were closed with absorbable sutures (Vicryl, 4–0, Ethicon GmbH). Animals received warmed saline (1 ml, s.c.), recovered and were kept in heated cages (30°C) for the rest of the experiments. For analgesia mice were treated with Carprofen (4 mg/kg, s.c.) directly after operation and next day with 12 h interval, if necessary longer. The bladder was manually emptied three times daily for the whole duration of the experiment.

### Echocardiographic Studies

Two-dimensional echocardiography imaging was performed under isoflurane anesthesia 1 day before and 7, 21, and 28 days post-injury/sham-surgery (dpi) using the Vevo 700 (VisualSonics, Toronto, Canada) equipped with scan head MS-400 with transmit frequency of 30 MHz. Anesthesia was induced with isoflurane 3% and maintained with (1.6 vol% isoflurane/air). Body temperature was maintained through a heating pad at 37°C. Images were acquired for further off-line analysis by an operator of Max Delbrück Center for Molecular Medicine Animal Phenotyping Platform blinded for genotype. LV end-diastolic and end-systolic diameter (LVIDd, LVIDs), EF, SV, and heart rate (HR) were determined by tracing of endocardial and epicardial borders and majors in end-diastole and in end-systole in the parasternal long axis view. LV end-diastolic and end-systolic posterior wall thickness (PWTd, PWTs) and anterior wall (interventricular septal wall) thickness (IVSd, IVSs) were determined by tracing in end-diastole and in end-systole in the parasternal short axis view. Following parameters were calculated: fractional shortening (FS, %) = 100 × ((LVIDd – LVIDs)/LVIDd); cardiac output (CO, ml/min) = SV × HR/1000; LV mass = 1.053 × ((LVIDd + LVPWd + IVSd)^3^ – LVIDd^3^).

### Quantitative RT-PCR

Hearts were halved so that the apex-facing part was processed further for RNA isolation and real-time qPCR analysis and the upper part was used for histology. For RNA isolation with Trizol and FastPrep beads the manufacturer’s instructions were followed. Reverse transcription was performed with 2 mg total RNA digested by incubation with 1 ml DNase I (Roche) using Moloney murine leukemia virus. Real-time PCR was performed with a QuantStudio 5 Real-Time PCR System (Thermo Fisher Scientific, Waltham, MA, United States) according to the manufacturers’ recommendations by using SYBR green. Each cDNA sample was tested in triplicate, and the expression level of each gene was normalized to the hypoxanthine–guanine phosphoribosyltransferase (Hprt) level. Following primers were used: *Agtr1a* forward: 5′-CCATTGTCCACCCGATGAAG-3′, *Agtr1a* reverse: 5′-TGC AGGTGACTTTGGCCAC-3′, *Agtr2* forward: 5′-CAATCTGGCT GTGGCTGACTT-3′, *Agtr2* reverse: 5′-TGCACATCACAGGT CCAA AGA-3′, *angiotensinogen* forward: 5′-TGTGACAGGGTG GAAGATGA-3′, reverse: 5′-CAGGCA GCTGAGAGAAACCT-3′, *Mas* forward: 5′-AGGGTGACTGACTGAGTTTGG-3′, re- verse: 5′-GAAGGTAAGAGGACAGGAGC-3′, *MAFbx* for- ward: 5′-AGTGAGGACCGGCTACTGTG-3′, reverse: 5′-GA TCAAACGCTTGCGAATCT-3′, *Murf1* forward: 5′-TGACAT CTACAAGCAGGAGTGC-3′, reverse: 5′-TCGTCTTCGTGTTC CTTGC-3′, *Hprt* forward: 5′-GTAATGATCAGTCAACGGGGG AC-3′, *Hprt* reverse: 5′-CCAGCAAGCTTGCAACCTTAACCA-3′. The fold change was determined using the 2^–Δ^ ^ΔC(t)^ method.

### HE and Picro Sirius Red Staining

Upper halves of the hearts were incubated in KCl for 10 min, fixed for 48 h in 4% paraformaldehyde in buffered saline (pH 7.4) at 4°C, washed, dehydrated, embedded in paraffin, sectioned at 5 μm with a rotary microtome (Microm; Thermo Fisher Scientific), placed on SuperFrost Plus slides (Thermo Fisher Scientific), and stored at RT until use. For picro Sirius red staining paraffin sections were deparaffinized, rehydrated, and stained by incubation with picro-Sirius red solution (0.1% Sirius red F3B in saturated aqueous solution of picric acid) for 1 h at room temperature. After two washes with acidified water (0.5% glacial acetic acid in tap water), sections were dehydrated in three changes of 100% ethanol, cleared using xylene, and coverslipped using Eukitt (ORSAtec GmbH, Bobingen, Germany). Four non-consecutive sections with hematoxylin–eosin (HE) and Sirius red staining per mouse were analyzed using an inverted microscope (BZ-9000; Keyence, Osaka, Japan). For immunostainings, pretreated paraffin sections were blocked for 30 min with 10% normal donkey serum in PBS, followed by incubation with the primary antibody polyclonal rabbit Murf1 (1:100, self-generated, Thomas Sommer, MDC, Germany) and goat anti-MAFxb (1:100; ab92281, Abcam) in PBS overnight at 4°C. The next day, sections were washed with PBS three times for 15 min and incubated with the secondary antibody solution in PBS for 2 h at room temperature. Sections were washed again three times with PBS and cover slipped using Vectashield (Vecta Laboratories, Burlingame, CA, United States) mounting medium containing DAPI. Images were taken using an inverted microscope (BZ-9000; Keyence, Osaka, Japan).

### Quantification of Cardiomyocyte Diameter

Wheat germ agglutinin (WGA) staining was performed by incubating heart sections with WGA + Al488 conjugate (1:300) (Thermo Fisher, Invitrogen, United States) for 20 h, washed three times with PBS for 10 min each and cover slipped using Vectashield (Vecta Laboratories, Burlingame, CA, United States) mounting medium containing DAPI. To evaluate cardiomyocyte diameter images were taken using an inverted microscope (BZ-9000) and 610-980 cardiomyocytes per group were analyzed using BZ-II Analyzer software (Keyence).

### Statistics

Results are presented as mean ± SEM. Data were analyzed statistically using Prism 5 software (GraphPad Software, San Diego, CA, United States) and SPSS (IMB Analytics). Echocardiography data were analyzed by three-way repeated measures (RM) ANOVA with time (pre, 7, 21, and 28 dpi) as one within-subject factor and genotype (WT, *Mas^–/–^*) and surgery (sham, T4-Tx) as two between subject factors. Once no significant three-factor interaction was detected, two-way RM ANOVA with Bonferroni posttest was performed. Heart weight (HW) data and gene expression data were analyzed by three-way ANOVA incorporating the following variables: genotype (WT, *Mas^–/–^*) and surgery (sham, T4-Tx) and time (1 month and 2 months post-surgery). Once no significant three-factor interaction was detected, one-way ANOVA with Bonferroni posttest was performed. Student’s *t*-test was applied for comparisons between individual pairs of means once normality was demonstrated. Significance for all tests was assumed when *p* < 0.05.

## Results

### Echocardiography: SCI Leads to Reduced LV Mass With Preserved FS and EF

**FIGURE 1:**
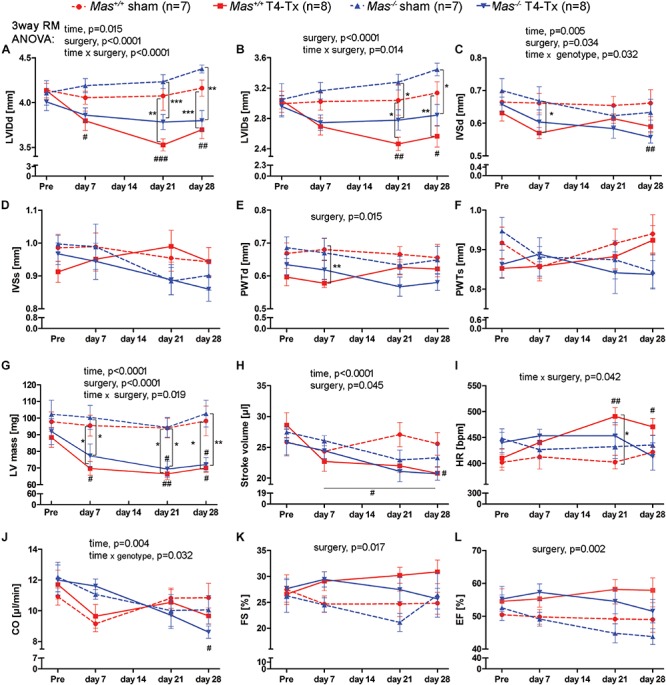
Echocardiography pre- and post-surgery in WT and *Mas^–/–^* sham-operated and T4-Tx mice. Structural parameters: **(A)** left ventricular internal diameter during diastole (LVIDd) and **(B)** LVID during systole (LVIDs), **(C)** interventricular septum thickness at diastole (IVSd) and **(D)** at systole (IVSs), **(E)** posterior wall thickness at diastole (PWTd), **(F)** at systole (PWTs), and **(G)** left ventricular mass (LV mass). Functional parameters: **(H)** stroke volume (SV), **(I)** heart rate, **(J)** cardiac output (CO), **(K)** fractional shortening (FS), and **(L)** ejection fraction (EF). Significant three-way repeated measures *ANOVA* results are presented above the graphs, two-way repeated measures ANOVA with *Bonferroni posttests* ^∗^*P* < 0.05, ^∗∗^*P* < 0.01, ^∗∗∗^*P* < 0.001 sham vs. T4-Tx; one-way *ANOVA* with *Bonferroni posttests* ^#^*P* < 0.05, ^##^*P* < 0.01, ^###^*P* < 0.001 pre vs. post-injury. Data are displayed as mean ± SEM.

Spinal cord injury caused a reduction in the LVIDd and LVIDs at 21 and 28 dpi vs. pre-injury and vs. sham in WT mice (Figures 1A,B, *p* < 0.05, two-way RM ANOVA). *Mas^–/–^* mice showed only a reduction in these indices when sham mice were compared (Figures 1A,B, *p* < 0.01, two-way RM ANOVA). IVSd was significantly reduced at 28 dpi vs. pre-injury in *Mas^–/–^* mice (Figure 1C, *p* < 0.01, one-way ANOVA) and at 7 dpi vs. sham in WT mice (*p* < 0.05, two-way RM ANOVA). PWTd at 7 dpi was in WT sham higher than in WT T4-Tx (Figure 1E). Consequently, LV mass was reduced at all time points following SCI (Figure 1G, *p* < 0.05, one-way ANOVA), except it was not significant at 7 dpi following SCI in *Mas^–/–^* mice. Progression of LV structural changes such as the reduction in LVIDd, LVIDs, LV mass in injured *Mas^–/–^* mice was not significantly different compared to injured WT mice (two-way RM ANOVA, interaction of time and genotype, all *p* > 0.0984). Although PWTd, PWTs, IVSd, and IVSs of *Mas^–/–^* mice seemed to be differentially regulated compared to WT (Figures 1C–F), two-way RM ANOVA of injured mice found no significant interaction of time and genotype for these parameters (all *p* > 0.0522). Functional changes included SV reduction post-SCI, HR increase at 21 and 28 dpi vs. pre-injury in the WT mice (Figures 1H,I, *p* < 0.05, one-way ANOVA) resulting in unchanged CO (Figure 1J). In contrast, SV was significantly reduced only at 28 dpi (two-way RM ANOVA, *p* < 0.05) and HR did not change significantly in the *Mas^–/–^* mice, but CO was reduced significantly at 28 dpi vs. pre-injury (*p* < 0.05, one-week ANOVA). Dynamics in SV was not different between injured *Mas^–/–^* and WT mice (two-way RM ANOVA, *p* = 0.5289), whereas dynamics of HR was (*p* = 0.0140). SCI caused no change in FS and EF (Figures 1K,L). They were lower in *Mas^–/–^* mice at 28 dpi, but difference to WT was not significant (two-way RM ANOVA, *p* = 0.1901). Three-way RM ANOVA performed on all the parameters revealed no significant interaction of surgery (sham, T4-Tx), genotype (WT, *Mas^–/–^*) and time (pre, 7, 21, and 28 dpi) (all *F*-values < 1.456, *p* > 0.234).

### Reduced Cardiac Weight and Cardiomyocyte Diameter Following SCI

**FIGURE 2:**
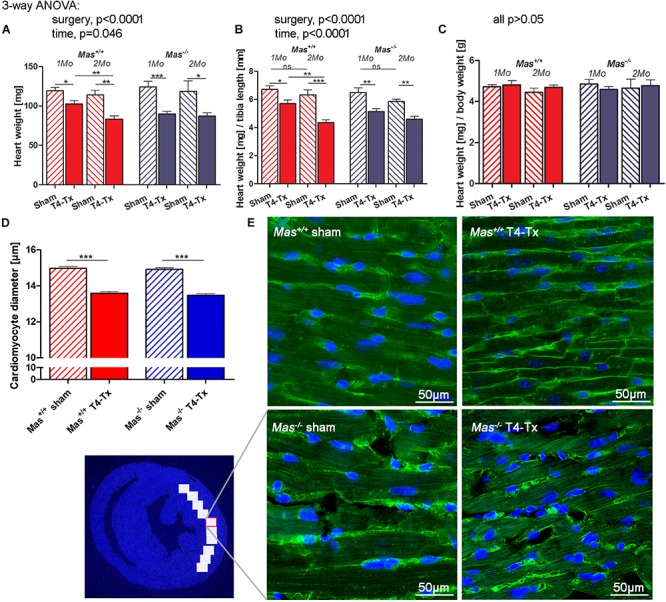
Cardiac atrophy and smaller cardiomyocyte diameter post-SCI. Heart weight (HW) **(A)** and its ratio with tibia length **(B)**, but not its ratio with body weight (HW/BW) **(C)** were reduced in injured mice compared to sham mice at 1 and 2 months post-SCI. **(D)** Cardiomyocyte diameter was smaller in injured mice. **(E)** Representative images of cardiomyocytes in a LV section stained with wheat germ agglutinin-Alexa Fluor 488 conjugate (green cell boundaries) and DAPI (blue nuclei). Scale bar is 50 μm. Three-way ANOVA significant main effects presented above the graphs **(A–C)**, ^∗^ (^∗∗^/^∗∗∗^)*P* < 0.05 (0.01/0.001) using Student’s *t*-test. Data are displayed as mean ± SEM.

Spinal cord injured mice had significantly smaller HW and HW/tibia length ratios at 1 month post-SCI, confirming the findings of echocardiography (Figures 2A,B, *p* < 0.05, one-week ANOVA). The decrease of HW was proportional to the body weight (BW) reduction as demonstrated by the unchanged HW/BW ratio (Figure 2C). HW and HW/tibia ratios remained significantly smaller in mice with T4-Tx at 2 months post-SCI compared to respective sham mice (Figures 2D,E, *p* < 0.05, one-way ANOVA). WT values at 2 months were significantly smaller vs. 1 month post-SCI (*p* < 0.01, Student’s *t*-test). The HW/BW ratio remained unchanged also at 2 months post-SCI. Histological analysis of the cardiomyocyte diameter at the mid-ventricular level revealed a significant decrease in the SCI groups vs. sham groups to similar extent in both genotypes (Figures 2D,E, *p* < 0.001, Student’s *t*-test).

### No Signs of Cardiac Pathology Post-SCI

**FIGURE 3:**
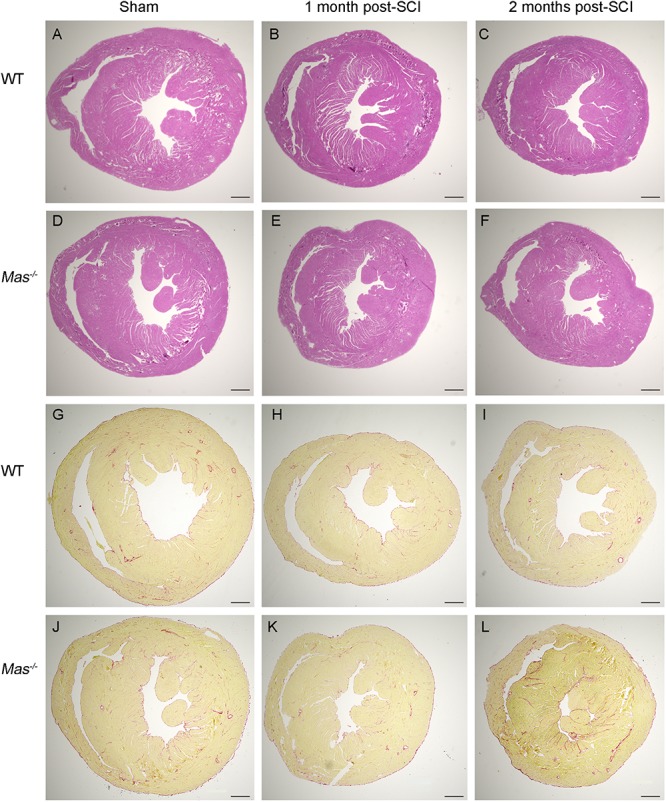
No histological alteration in the heart post-SCI. Hematoxylin-eosin (**A–F)** and picro Sirius red staining **(G–L)** of heart paraffin sections from sham-operated and spinal cord-injured WT and *Mas^–/–^* mice 1 and 2 months post-SCI. Scale bar: 500 μm.

The myocardium of SCI mice presented normal histology, absence of cardiomyocyte necrosis, infiltration of cells (Figures 3A–F). We found no interstitial as well as perivascular fibrosis assessed by picro Sirius red staining at 1 and 2 months post-SCI (Figures 3G–L).

### Atrogene and RAS Expression Post-SCI

**FIGURE 4:**
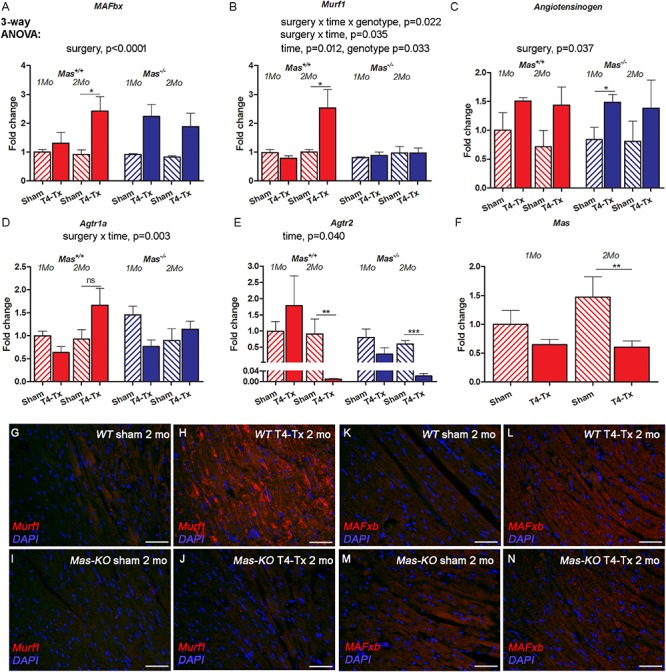
Gene expression of atrogenes and RAS members in the heart of 1-month and 2 months post-SCI in WT and *Mas^–/–^* mice. Atrogene *MAFbx* was upregulated in WT mice at 2 months **(A)**, whereas *Murf1* remained unchanged in *Mas^–/–^* **(B)**. *Angiotensinogen* was upregulated significantly in *Mas^–/–^* mice at 1 months post-SCI **(C)**, *Agtr1a* was not significantly regulated **(D)**, *Agtr2* was downregulated significantly in *Mas*^+/+^ and *Mas^–/–^* mice at 2 months post-SCI **(E)** and *Mas* was downregulated in control *Mas*^+/+^ mice at 2 months post-SCI **(F)**. Three-way ANOVA significant interactions and main effects presented above the graphs, ^∗^*p* < 0.05, ^∗∗^*p* < 0.01, ^∗∗∗^*p* < 0.005 *Student’s t-test.* Representative images of immunostaining of Murf1 **(G–J)** and MAFxb **(K–N)** in the heart in sham and injured mice 2 months post-SCI. Scale bar 50 μm.

Cardiac atrophy is usually accompanied by the upregulation of atrogenes such as Muscle Atrophy F-box (*MAFbx*) and RING-finger protein-1 (*Murf1*). Gene expression of *MAFbx* was upregulated, but this difference reached significance only at 2 months post-SCI in WT mice (Figure 4A, *p* < 0.05, Student’s *t*-test). *Murf1* showed only a trend for upregulation at 2 months post-injury in WT mice (Figure 4B). *Angiotensinogen* was upregulated at 1 month post-SCI in *Mas^–/–^* mice (Figure 4C, *p* < 0.05, Student’s *t*-test). Ang II receptor 1a (*Agtr1a*) was not significantly regulated (Figure 4D), whereas Ang II receptor 2 (*Agtr2*) was downregulated at 2 months post-SCI in *Mas^–/–^* mice (Figure 4E, *p* < 0.001, Student’s *t*-test). *Mas* was downregulated 2 months post-SCI in WT mice (Figure 4F, *p* < 0.01, Student’s *t*-test). Immunohistochemistry of Murf1 and MAFbx showed increased immunofluorescence in injured mice compared to sham at 2 months post-SCI (Figures 4G–N, respectively).

## Discussion

Our study is the first to examine the effect of SCI on murine heart using echocardiography, histology, and gene expression analysis in WT and *Mas*-deficient mice. We found that T4-Tx SCI caused persistent cardiac atrophy with significant reduction in LV mass, HW, and cardiomyocyte diameter at 1 month post-injury, reduced HW at 2 months post-SCI, preserved EF and FS, no fibrosis nor pathological signs in the hearts of injured mice at 1 and 2 months post-SCI. Differential effects of SCI in WT compared to *Mas^–/–^* mice included reduced IVSd and PWTd at 7 dpi vs. sham, as well as increased HR at 21 and 28 dpi in injured WT mice, whereas *Mas^–/–^* mice had reduced CO at 28 dpi.

The impact of Mas-deficiency on heart following high-thoracic SCI was modest, taking into account that this receptor plays positive role in many cardiac conditions (Bader, 2013). For example, Mas is an important regulator of vascular tonus being involved in the vasodilator effect of Ang-(1–7), bradykinin, acetylcholine (Peiro et al., 2007; Peiró et al., 2013), and estradiol (Sobrino et al., 2010, 2017) which is beneficial in the hypertensive situation. After T4-Tx, however, drastic hypotension occurs in mice (Jarve et al., 2018), due to the loss of sympathetic vasoconstrictor input to the vascular beds below the injury. In this situation, Mas-deficiency rather than Mas-mediated vasodilation might be advantageous. Indeed, Mas-deficient mice have less severe hypotension in the acute phase of SCI (Jarve et al., 2018). This favorable effect might occur because of higher vascular tonus and/or higher CO in *Mas^–/–^* mice. In this study, CO of *Mas^–/–^* mice was higher at acute phase compared to WT mice (*t*-test, *p* = 0.0481 at 7 dpi) indicating that CO might contribute to this effect. In contrast, in chronic phase of SCI (28 dpi), CO of *Mas^–/–^* mice decreased significantly meaning that at this stage higher vascular tonus in these mice must exist as hypotension was comparable between the phenotypes. This is not accompanied by hyperreactivity of the vasculature to phenylephrine and Ang II in *Mas^–/–^* mice or in its isolated mesenteric vessels (Jarve et al., 2018). CO depends on SV and HF. As SV was similarly decreased at 28 dpi in both genotypes, lower CO at 28 dpi in *Mas^–/–^* mice is due to lower HF in these mice, a finding which matches well with our previous telemetry study (Jarve et al., 2018).

### Echocardiography Data in Mouse SCI Model Is in Good Agreement With Clinical Data

Our findings of cardiac atrophy (LV mass reduction ca. 22% in the present study and 25% in patients) with preserved function agrees well with SCI patient data (Williams et al., 2019), in which the atrophy is often considered as a physiological adaption, e.g., to preserve function (Sharif et al., 2017). Also a pre-clinical study in rats at 35 dpi agrees well with our data (West et al., 2014). The same group observed however reduced EF and FS at 3 months post-SCI (Poormasjedi-Meibod et al., 2019), which we cannot exclude to happen also in our model at a later time point. Cardiac atrophy occurs also in healthy persons after 2 weeks of bed-rest and reaches a 15% decrease in LV mass index after 12-week-bed rest (Perhonen et al., 2001). Even in some proportion of cachexia patients significant loss of LV mass was not associated with specific cardiac abnormalities (Florea et al., 2002) indicating that it is possible to have atrophy and maybe altered but not compromised function of the heart. Furthermore, in our T4-Tx model, the heart would be deprived from at least a proportion of sympathetic innervation to the LV (Strack et al., 1988), also resulting in loss of trophic support and consequently leading to cardiac atrophy (Zaglia et al., 2013), which was not accompanied by dysfunction.

Could activation of the RAS contribute to cardiac atrophy? The role of Ang II and its receptor Agtr1a in skeletal muscle atrophy is well established (Burks et al., 2011; Cabello-Verrugio et al., 2012). Mas activation on the other hand counteracts skeletal muscle atrophy induced by Ang II (Cisternas et al., 2015), disuse (Morales et al., 2016), and cancer (Murphy et al., 2019). SCI with profound hypotension activates the RAS as its main mediator Ang II is significantly elevated (Frankel et al., 1972; Groothuis et al., 2010; Poormasjedi-Meibod et al., 2019) and expression of its receptor Agtr1a is increased in the heart post-SCI (Poormasjedi-Meibod et al., 2019). Together with our findings of elevated *angiotensinogen* mRNA post-SCI (at 1-month post-SCI in *Mas^–/–^* mice) and a trend for *Agtr1a* upregulation (at 2 months post-SCI in WT mice) a potential atrophy-inducing environment might be present in the myocard, on top of cardiac unloading and loss of cardiac sympathetic innervation. We have previously demonstrated that *Mas^–/–^* mice present more severe atrophy of the gastrocnemius and plantaris muscles at 28 days post-T4-Tx (Järve et al., 2019). This prompted us to assume in this study that *Mas^–/–^* mice would suffer more from cardiac atrophy post-SCI. However, our results showed that absence of Mas is not aggravating cardiac atrophy. This would not necessarily exclude the possibility that an infusion of Ang-(1–7) would have a positive effect on cardiac atrophy. However, in the light of the downregulation of *Mas* determined in this study a prospective vasodilation effect of such treatment post-SCI would be difficult to achieve.

We did not observe any fibrosis in the hearts of injured mice nor did we find any other histological indices of end-organ damage in the heart. For example, long-term unloading after heterotopic heart transplantation was associated with decreased function and increased fibrosis (Oriyanhan et al., 2007). It has been suggested that the cardiac unloading itself might produce cardiac remodeling post-SCI (e.g., increased fibrosis) (Lujan and Dicarlo, 2014). Further studies with longer time points could be necessary to reveal this effect.

We used only female mice in our study. It is also important to analyze male mice, because hormonal systems such as estradiol influence the vascular system and thus the effects we observed in the knockout mice could be influenced by this. It has been shown that estradiol in physiological levels can cause vasodilation in the presence of Mas (Sobrino et al., 2017). Conversely, in *Mas^–/–^* mice probably this vasodilation effect of estradiol is impaired.

It was not surprising to find some regulation in the ubiquitin/proteasome system (UPS) genes. It would be necessary to investigate autophagy in future studies, as it is involved in cardiac atrophy following SCI (Poormasjedi-Meibod et al., 2019) and is dominating in unloading-induced atrophy, whereas activation of UPS is most important in starvation−induced cardiac atrophy (Cao et al., 2013).

## Conclusion

T4-Tx SCI leads to significant atrophy of the murine heart. In light of preserved EF and FS as well as in the absence of signs of end-organ damage, there is no reason to consider the murine heart compromised or dysfunctional at 1-month post-SCI, at least in rest. Furthermore, lack of *Mas*, is not detrimental to cardiac structure or function in the early chronic phase of SCI. Future studies with longer time frame could show how the changes progress over time and whether other members of the RAS are involved.

## Data Availability Statement

All datasets generated for this study are included in the article/Supplementary Material.

## Ethics Statement

The animal study was reviewed and approved by the Animal Care and Use Committee from Berlin LAGeSo.

## Author Contributions

AJ conceptualized the study, performed the experiments, analyzed the data, and wrote the manuscript. FQ, MT, and SS performed the experiments and analyzed the data. NA revised the manuscript. MB helped by planning the experiments and manuscript revision.

## Conflict of Interest

The authors declare that the research was conducted in the absence of any commercial or financial relationships that could be construed as a potential conflict of interest.
