# Supplementary material for: Angiotensin-(1–7) Receptor Mas Deficiency Does Not Exacerbate Cardiac Atrophy Following High-Level Spinal Cord Injury in Mice
Source: Front Physiol. 2020 Mar 12;11:203. doi: 10.3389/fphys.2020.00203 (PMC7080696; doi:10.3389/fphys.2020.00203)
Supplement: Supplementary file 1 [file Table_1.DOCX]

**Supplementary Data**

**
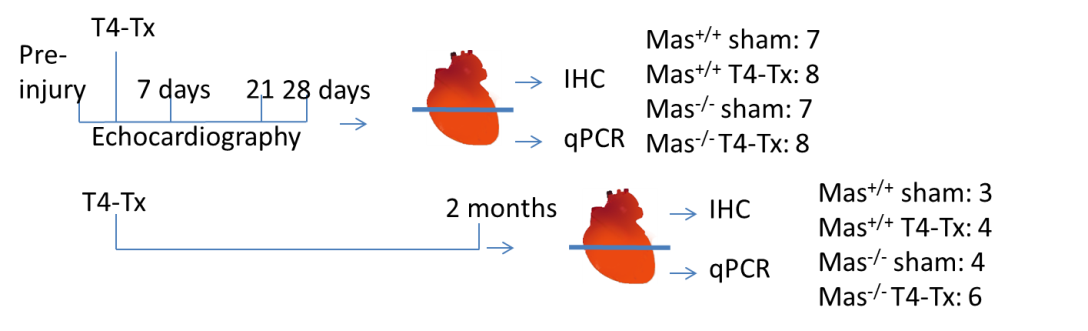
**

**Supplementary Fig. 1. Experimental plan.** There were 2 groups of mice, one group received echocardiography and was sacrificed one month post-SCI, the other group was sacrificed 2 months post-SCI.
